# Methacholine hyperresponsiveness in mice with house dust mite‐induced lung inflammation is not associated with excessive airway constriction ex vivo

**DOI:** 10.1113/EP092522

**Published:** 2025-03-28

**Authors:** Andrés Rojas‐Ruiz, Magali Boucher, Cyndi Henry, Louis Gélinas, Rosalie Packwood, Percival Graham, Jorge Soliz, Ynuk Bossé

**Affiliations:** ^1^ Institut Universitaire de Cardiologie et de Pneumologie de Québec (IUCPQ) – Université Laval Québec Quebec Canada; ^2^ SCIREQ Inc. Montreal Quebec Canada

**Keywords:** animal model, methacholine response, smooth muscle contraction

## Abstract

The role of excessive airway constriction in the hyperresponsiveness to nebulized methacholine in mice with experimental asthma is still contentious. Yet, there have been very few studies investigating whether the increased in vivo response to methacholine caused by experimental asthma is associated with a corresponding increase in ex vivo airway constriction. Herein, the responses to nebulized methacholine in vivo and airway constriction in lung slices ex vivo were studied in 8‐ to 10‐week‐old male mice of two strains, BALB/c and C57BL/6. Experimental asthma was induced by administering house dust mites (HDM) intranasally, once daily, for 10 consecutive days. Complementary ex vivo studies were conducted with excised tracheas to measure and compare isometric force. As expected, the in vivo response to methacholine, and especially the hyperresponsiveness caused by HDM, was greater in BALB/c than in C57BL/6 mice. In contrast, there were no differences in maximal airway constriction between mouse strains, and the hyperresponsiveness to nebulized methacholine caused by HDM in both mouse strains was not associated with a corresponding increase in ex vivo airway constriction. The experiments with excised tracheas demonstrated no differences in isometric force between strains and between mice with and without experimental asthma. It is concluded that the hyperresponsiveness to nebulized methacholine in an acute mouse model of asthma induced by repeated HDM exposures is not associated with excessive airway constriction ex vivo.

## Abstract (highlights)

- **What is the central question of this study?**
  In this study, we investigated the association between the in vivo response of the respiratory system to nebulized methacholine and the ex vivo responsiveness of airways in two mouse strains with and without experimental asthma induced by repetitive intranasal exposures to house dust mites. The ex vivo assays included measurements of airway constriction in lung slices and measurements of isometric force with excised tracheas.
- **What is the main finding and its importance?**
  Although striking differences in the in vivo response to methacholine were observed between mouse strains and between mice with and without experimental asthma, these changes were not matched by corresponding changes in ex vivo airway responsiveness.

## 1 INTRODUCTION

Hyperresponsiveness to inhaled methacholine is a hallmark of human asthma (Nair et al., 2017). It is thus generally accepted that an appropriate animal model of asthma should exhibit this feature. As a result, most, if not all, mouse models of asthma are hyperresponsive to nebulized methacholine. It is often assumed that this hyperresponsiveness caused by experimental asthma is mediated, at least in part, by excessive airway constriction. Yet, there is a dearth of studies investigating whether the increased in vivo response to methacholine in specific models of asthma is associated with a corresponding increase in ex vivo airway constriction. In the present study, we investigated the association between the in vivo response to nebulized methacholine and the ex vivo constriction of airways in two mouse strains with and without experimental asthma.

BALB/c and C57BL/6 were chosen not only because they are the most widely used mouse strains for modelling human asthma (Carroll et al., 2023), but also because their innate response to nebulized methacholine (i.e., in the absence of experimental asthma) is vastly different, with BALB/c mice being more responsive than C57BL/6 mice (Berndt et al., 2011; Duguet et al., 2000; Held & Uhlig, 2000; Leme et al., 2010; Levitt & Mitzner, 1989). The BALB/c mice are also generally more susceptible than C57BL/6 mice to acquire hyperresponsiveness (i.e., induced by experimental asthma) (Adler et al., 2004; Boucher et al., 2021; De Vooght et al., 2010; Evans et al., 2015; Ewart et al., 2000; Gueders et al., 2009; Hirota, Ask et al., 2009; Kelada et al., 2011; Kenyon et al., 2003; Koya et al., 2006; Li et al., 2017; Sahu et al., 2010; Shinagawa & Kojima, 2003; Takeda et al., 2001; Van Hove et al., 2009; Zhang et al., 1997; Zhu & Gilmour, 2009). Given that many early studies, conducted with both mice without experimental asthma (Weinmann et al., 1990) and non‐asthmatic humans (Armour, Black et al., 1984; Armour, Lazar et al. 1984; Cerrina et al., 1986; de Jongste et al., 1988; Roberts et al., 1987; Taylor et al., 1985; Thomson, 1987), have demonstrated that the level of airway responsiveness in vitro rarely matches the degree of in vivo responsiveness, it is hypothesized that the in vivo hyperresponsiveness caused by experimental asthma will not be associated with changes in ex vivo airway constriction in either mouse strain.

## 2 MATERIALS AND METHODS

### 2.1 Ethical approval

All methods were approved by the Committee of Animal Care of Université Laval, following the guidelines from the Canadian Council on Animal Care (2020‐652‐4). The research also adhered to *Experimental Physiology*’s policies regarding animal experiments.

### 2.2 Mice

Forty‐eight male BALB/c mice (Charles River, Saint‐Constant, QC, Canada) and 48 male C57BL/6 mice (The Jackson Laboratory, Bar Harbor, ME, USA) were studied between the ages of 8 and 10 weeks. Males were used because they have more airway smooth muscle (ASM) (Gill et al., 2023) and their response to nebulized methacholine is markedly greater than that of females (Card et al., 2006; Gill et al., 2023). They were provided with food and water ad libitum.

### 2.3 Experimental model of asthma

Experimental asthma was induced as previously described (Boucher et al., 2021; Rojas‐Ruiz et al., 2024). Briefly, mice were exposed to either 25 µL of 0.9% sterile saline or 25 µL of 2 mg/mL house‐dust mite (HDM) extract (*Dermatophagoides pteronyssinus*, lot number 360923; Greer Laboratories, Lenoir, NC, USA) diluted in 0.9% sterile saline to induce allergic lung inflammation. The endotoxin concentration was 47.3 EU/mg of HDM extract. The exposure occurred once daily via an intranasal instillation for 10 consecutive days under isoflurane anaesthesia. All measurements were made the day after the last exposure.

### 2.4 Mechanical ventilation

Twelve mice per group were anaesthetized by administering ketamine (100 mg/kg) and xylazine (10 mg/kg) (Dechra Veterinary Products, Pointe‐Claire, Canada) intraperitoneally. They were then tracheotomized and connected to the flexiVent (FX Module 2, SCIREQ, Montreal, QC, Canada) through an 18‐gauge cannula in a supine position. To prevent leakage, a surgical thread was used to secure and seal the trachea on the cannula. They were ventilated mechanically with air at a tidal volume of 10 mL/kg with an inspiratory‐to‐expiratory time ratio of 2:3 at a breathing frequency of 150 breaths/min and with a positive end‐expiratory pressure of 3 cmH_2_O. Once the ventilation was underway, muscle relaxation was induced by injecting 100 and 300 µL of pancuronium bromide (0.12 mg/mL) intramuscularly and intraperitoneally, respectively, to avoid spontaneous breathing during the experiments. Heart rate was monitored continuously by electrocardiography throughout the experiment to ensure proper anesthesia.

### 2.5 The in vivo methacholine challenge

While on the flexiVent, mice were subjected to a multiple‐concentration challenge with methacholine. The concentrations used were 0, 10 and 30 mg/ml, all diluted in PBS. They were nebulized at 5 min intervals. For each concentration, the nebulizer for small particle size (Aeroneb Lab, Aerogen, Galway, Ireland) was operating for a duration of 10 s at a duty cycle of 50% under regular ventilation. Two lung recruitment manoeuvres to 30 cmH_2_O were performed before the start of the methacholine challenge and after each subsequent concentration to avoid alveolar collapse, in line with an optimized protocol of mechanical ventilation (Reiss et al., 2011).

To monitor the changes in respiratory mechanics during the methacholine challenge, two short oscillometric perturbations of small amplitude, called the SnapShot‐150 (1.25 s) and the Quick Prime‐3 (3 s) were used. The former consists of a single sine‐wave oscillation at 2.5 Hz that allows resistance (*R*_rs_) and elastance (*E*_rs_) of the respiratory system to be calculated based on the linear single‐compartment model (Bates, 2009). The latter is an oscillometric perturbation composed of an input flow signal made of 13 sine waves of mutually prime frequencies with different amplitudes and phases, allowing the impedance spectrum of the respiratory system from 1 to 20.5 Hz to be calculated (Bates et al., 2011). The impedance spectrum was then fitted to the constant phase model for computing three parameters (Hantos et al., 1992). One is airway resistance (*R*_aw_), which reflects the resistance to airflow in conducting airways, although it can sometimes be influenced by the chest wall (Hirai et al., 1999; Ito et al., 2007; Sudy et al., 2019). Another one is tissue resistance (*G*), which reflects the tissue resistance of the lung and the chest wall (Hirai et al., 1999; Ito et al., 2007; Sudy et al., 2019) but is also sensitive to small airway narrowing heterogeneity (Lutchen et al., 1996). The final one is tissue elastance (*H*), which reflects the elastance of the whole lung and is thus sensitive to both the accessible (i.e., reachable from the mouth) volume of the lung and the tissue stiffness of the lung and the chest wall (Hirai et al., 1999; Sudy et al., 2019). The hysteresivity (η), which is the ratio of *G* over *H*, was also determined.

The SnapShot‐150 and the Quick Prime‐3 were each actuated twice at baseline in an alternating fashion. They were then each actuated 10 times, again in an alternating fashion, after each methacholine concentration, starting 10 s after the nebulization. Eight seconds of tidal breathing was intercalated between each actuation to avoid desaturation. The changes in oscillometric readouts described above, namely *R*_rs_, *E*_rs_, *H*, *G*, *R*_aw_ and η, from their baseline values to their peak values following each concentration of methacholine were used to trace the concentration–response curve. The methacholine response was then quantified by measuring the area under the curve for each oscillometric readout.

### 2.6 Euthanasia

The mice undergoing the methacholine challenge, representing half of the mice, were killed by exsanguination after the last flexiVent measurement. The other half were killed by lethal intraperitoneal injection of ketamine (200 mg/kg) and xylazine (20 mg/kg). The latter mice were required for collection of tracheas and lungs for the ex vivo assays (described below). It was not possible to conduct the in vivo and ex vivo experiments on the same mice, because the cannulation required for the flexiVent alters tracheal contraction, and the in vivo exposure to methacholine compromises airway constriction in lung slices because it prevents a smooth and homogeneous filling of the lungs with agarose.

### 2.7 Wet lung weight

The lungs were surgically removed, cleaned, and weighed using a laboratory digital scale (Mettler Toledo, Mississauga, ON, Canada).

### 2.8 Histology

Histology was performed on the left unilobed lung in a total of 12 mice per group. The lobe was excised and immersed in formalin for 24 h for fixation. The tissue was subsequently dehydrated by substituting the formalin with increasing concentrations of ethanol. The lobe was then embedded in paraffin and cut transversely into 5‐µm‐thick sections. Sections were deposited on microscope slides, stained, and scanned with a NanoZoomer Digital scanner (Hamamatsu Photonics, Bridgewater, NJ, USA) at ×40 magnification.

Histological alterations seen in this specific model of experimental asthma were characterized previously (Boucher et al., 2021; Gill et al., 2023; Rojas‐Ruiz et al., 2024). It was repeated herein to confirm the establishment of allergic lung inflammation. Lung sections stained with Haematoxylin and Eosin were used to quantify tissue infiltration with inflammatory cells. Sixteen non‐overlapping photomicrographs (1440 × 904 pixels) from four non‐contiguous lung sections were scored blindly from zero (no inflammation) to five (very severe inflammation). Lung sections stained with Periodic Acid–Schiff (PAS) and Alcian Blue were used to count the number of goblet cells. All airways cut transversely in four non‐contiguous lung sections were analysed, representing 9–38 airways per mouse (average of 23.2 ± 6.8). Lung sections stained with Masson Trichrome were used to quantify the content of ASM and the thickness of the epithelium. All airways cut transversely in four non‐contiguous lung sections were analysed, representing 10–34 airways per mouse (average of 18 ± 5.6). The content of ASM in each airway was calculated by measuring the area occupied by the muscle divided by the square of its basement membrane perimeter (Bullone et al., 2014). The thickness of the epithelium was also analysed for the same airways by measuring the area occupied by the epithelium divided by the basement membrane perimeter.

### 2.9 Constriction of airways in lung slices

Airway constriction in lung slices was measured in 12 mice per group. After euthanasia, thoracotomy was performed, and the chest was opened. While the lungs were still resting inside the thorax, the right lobe was ligated, and a tracheotomy was performed in the lower part of the trachea. Then, 350 µL of agarose (catalogue no. 1613112; Bio‐Rad Laboratories, Redmond, WA, USA) at 40°C was infused through the trachea using an 18‐gauge syringe. The left lung was then plunged into ice‐cold Hank's balanced salt solution (HBSS) for 30 min to polymerize the agarose. Slices of 200 µm thick were cut in the sagittal orientation using the Compresstome VF‐310 (Precisionary Instruments, Ashland, MA, USA). Slices were selected more precisely in the middle part of the lobe, avoiding the first and last 20% edges. Finally, lung slices were placed into a six‐well plate containing Dulbecco's modified Eagle's medium supplemented with 1% penicillin, streptomycin and amphotericin B and kept at 37°C for 24 h. Four slices were put into each well.

Airway constriction was measured by the physioLens (SCIREQ, Montreal, QC, Canada) as recently described (Boucher et al., 2024). This was done one well at a time. The first step consisted of removing the Dulbecco's modified Eagle's medium and washing the slices twice with warm HBSS using a pipette. The slices were then fixed in the middle of the well with a SCIREQ slice holder (physioMesh) in 6 mL of warm HBSS and transferred to the physioLens. The experimenter subsequently selected on the computer screen all clearly visible airways with a perpendicular orientation in relationship to the field of view (i.e., circular airways), with no apparent damage and with clear beating of the cilia. The following steps were then executed automatically by the physioLens. First, the lung slices were washed again with HBSS and left untouched for 3 min. This washing step allowed many airways to dilate fully. Then, the smooth muscle was stimulated to contract with methacholine dissolved in HBSS warmed to 37°C at four incremental concentrations: 10^−7^, 10^−6^, 10^−5^ and 10^−4^ M. Each concentration was replaced at 3 min intervals. Images of the luminal area were collected in all preselected airways within the well every 1 min. Constriction, expressed as a percentage reduction in relationship to the initial luminal area (the one at the end of the last washing step with HBSS), was also calculated automatically. The maximal constriction obtained during the 3 min of recording at each methacholine concentration was used to generate the concentration–response curve. These procedures were then repeated for each of the other wells.

### 2.10 Contraction of excised tracheas

Tracheal contraction was measured in 12 mice per group (in the same mice as for the airway constriction in lung slices). The whole trachea was collected and immersed in Krebs solution (pH 7.4, 111.9 mM NaCl, 5.0 mM KCl, 1.0 mM KH_2_PO_4_, 2.1 mM MgSO_4_, 29.8 mM NaHCO_3_, 11.5 mM glucose and 2.9 mM CaCl_2_). It was then mounted horizontally in a 5 mL organ bath containing Krebs solution maintained at 37°C. The preparation was coupled to a force transducer (Harvard Apparatus, St Laurent, QC, Canada) for measuring isometric force. A resting distending force of 5 mN was applied. Before the contractile assays, the trachea was subjected to a period of conditioning, during which time it was stimulated to contract repeatedly for 5 min at 10 min intervals with 10^−5^ M methacholine until a reproducible force was recorded.

Cumulative concentration–response curves were then generated with methacholine and KCl. Methacholine was added in logarithmic increments at 5 min intervals from 10^−7^ to 10^−4^ M. The concentration of KCl was doubled at 5 min intervals from 20 to 160 mM. The peak force obtained at each concentration was used to generate the concentration–response curves. The trachea was washed repeatedly with fresh Krebs for ≥30 min between methacholine and KCl. To measure potency, the EC_50_ (the concentration causing half of the maximal response) was also calculated by fitting the data by the least‐squares method to the following equation: *y* = bottom + *x* ^(top − bottom)/(EC50 +^ *^x^* ^)^.

### 2.11 Data analysis

Two‐way ANOVAs were used to assess the effect of experimental asthma, the mouse strain and their interaction on each measured readout. When the interaction was significant, it was followed by Sidak's multiple comparisons test specifically to compare mice with and without experimental asthma in each mouse strain. Three‐way ANOVAs were also used for the ex vivo contractile assays to assess the effect of experimental asthma, the mouse strain, the concentration–response to methacholine and their interactions. Statistical analyses were performed with Prism (v.10.2.1; GraphPad, San Diego, CA, USA).

## 3 RESULTS

**FIGURE 1:**
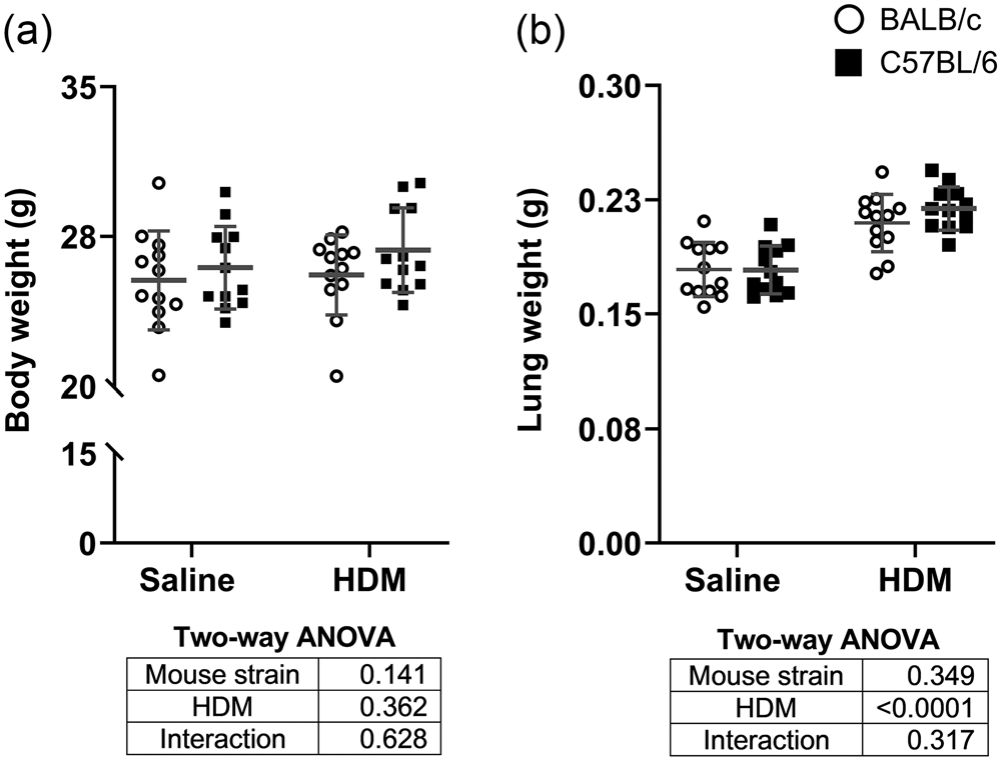
Body weight (a) and total lung weight (b) in BALB/c mice (open circles) and C57BL/6 mice (filled squares) that were exposed to either saline or HDM. Individual results are presented, together with means ± SD. Results of two‐way ANOVAs are provided underneath the graphs. *n* = 12 mice per group. Abbreviation: HDM, house dust mite.

The body weight was similar in both mouse strains and was not affected by HDM (Figure 1a). The wet weight of the lungs was also not different between BALB/c and C57BL/6 mice. However, HDM‐exposed mice had heavier lungs (*P *< 0.0001; Figure 1b).

**FIGURE 2:**
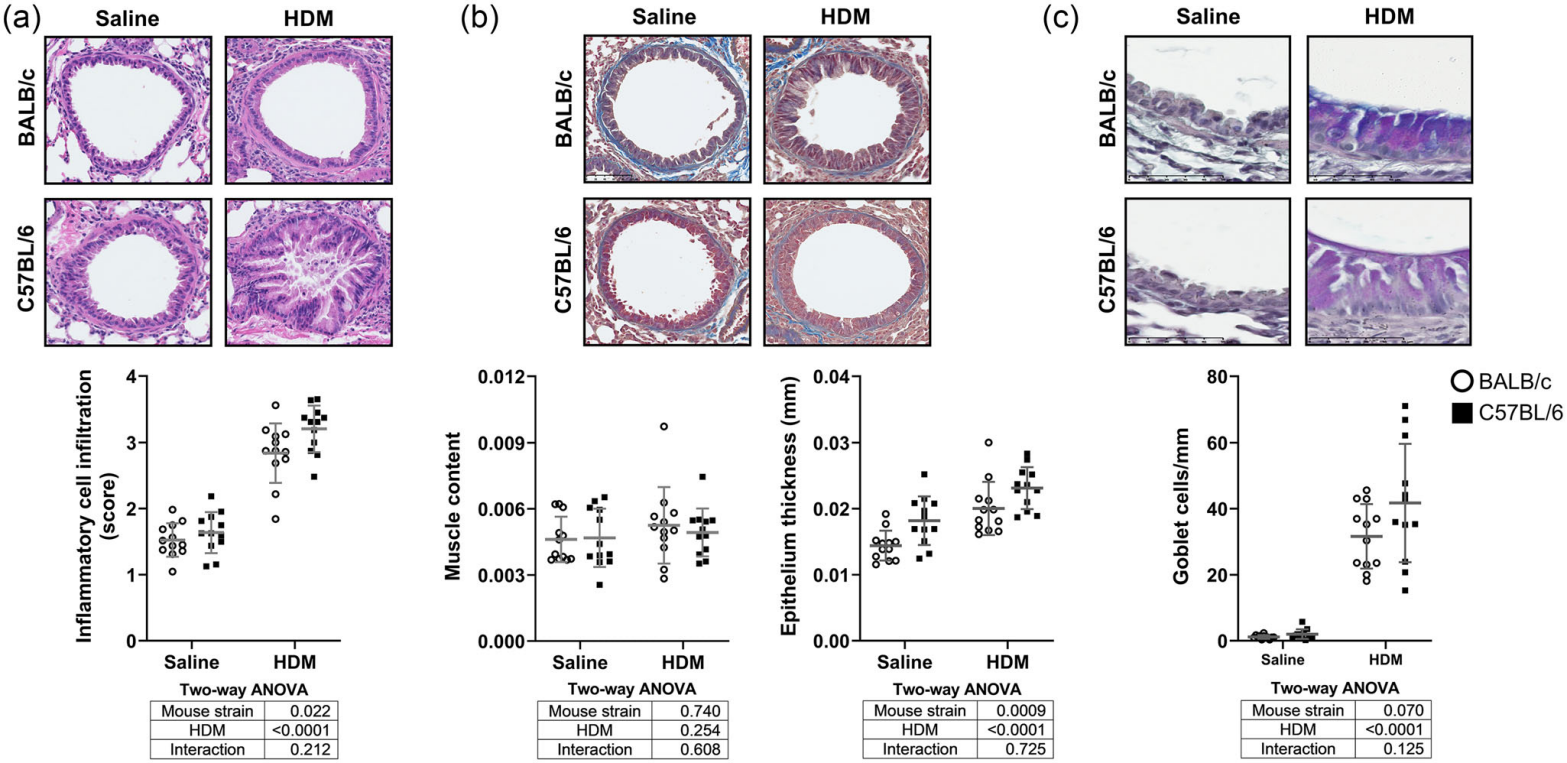
Histological analyses on lung sections of BALB/c mice (open circles) and C57BL/6 mice (filled squares) that were exposed to either saline or HDM. Representative images are shown at the top, and individual results, together with means ± SD, are shown at the bottom. Lung sections were stained with Haematoxylin and Eosin to quantify tissue infiltration with inflammatory cells (a), Masson Trichome to quantify the content of airway smooth muscle and the thickness of the epithelium (b) or Periodic Acid–Schiff and Alcian Blue to quantify the amount of goblet cells in the airway epithelium (c). Results of two‐way ANOVAs are provided underneath the graphs. *n* = 12 mice per group. Abbreviation: HDM, house dust mite.

Lung tissue alterations caused by HDM were confirmed by histology (Figure 2). Lungs of mice exposed to HDM exhibit a greater tissue infiltration with inflammatory cells (Figure 2a), no change in the content of ASM (Figure 2b), an increased thickness of the airway epithelium (Figure 2b) and a higher number of goblet cells (Figure 2c). The histological alterations caused by HDM were similar between the two mouse strains; however, C57BL/6 mice exhibited a higher tissue infiltration with inflammatory cells and a thicker epithelium compared with BALB/c mice.

**FIGURE 3:**
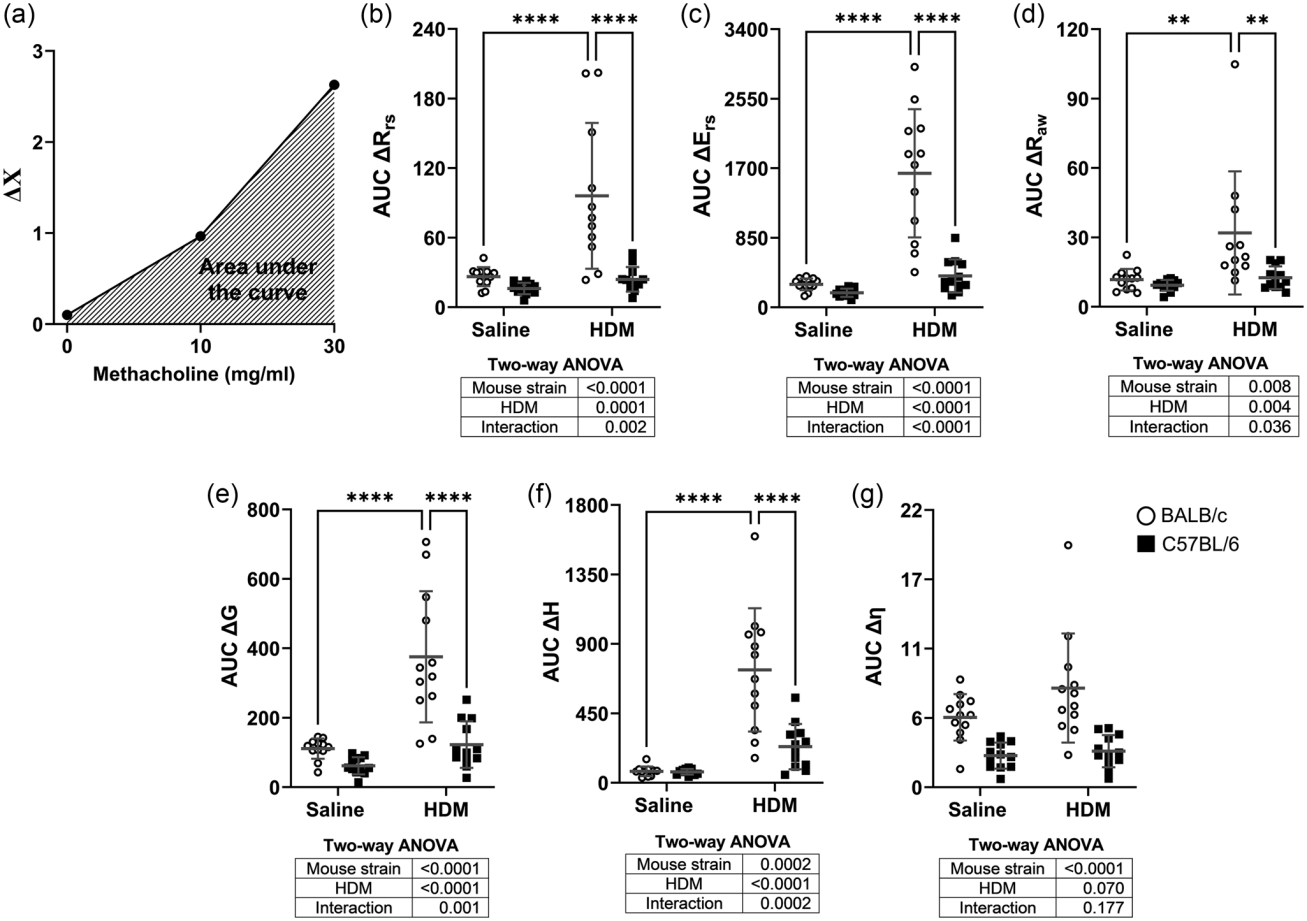
The response to nebulized methacholine in BALB/c mice (open circles) and C57BL/6 mice (filled squares) that were exposed to either saline or HDM. (a) The response was quantified by measuring the AUC of the changes (Δ) in each parameter of respiratory mechanics caused by methacholine. (b–g) Individual results are shown, together with means ± SD for respiratory system resistance (Δ*R*_rs_; b), respiratory system elastance (Δ*E*_rs_; c), airway resistance (Δ*R*_aw_; d) tissue resistance (Δ*G*; e), tissue elastance (Δ*H*; f) and hysteresivity (η; g). Results of two‐way ANOVAs are provided underneath the graphs. When the interaction was significant, Sidak's multiple comparisons test was conducted, and asterisks indicate significant differences (^**^ *P *< 0.01 and ^****^ *P *< 0.0001). *n* = 12 mice per group. Abbreviations: AUC, area under the curve; HDM, house dust mite.

The response to nebulized methacholine is depicted in Figure 3. As expected, HDM enhanced the methacholine response irrespective of the parameter used to measure the response (∆*R*_rs_, ∆*E*_rs_, ∆*H*, ∆*G*, ∆*R*_aw_ and ∆η; Figure 3b–g). The mouse strain also had a significant effect on all parameters except ∆η, indicating that C57BL/6 mice are less responsive in vivo than BALB/c mice. There were also significant interactions between HDM and the mouse strain for all parameters except ∆η, suggesting that the in vivo hyperresponsiveness caused by HDM was greater in BALB/c than in C57BL/6 mice.

**FIGURE 4:**
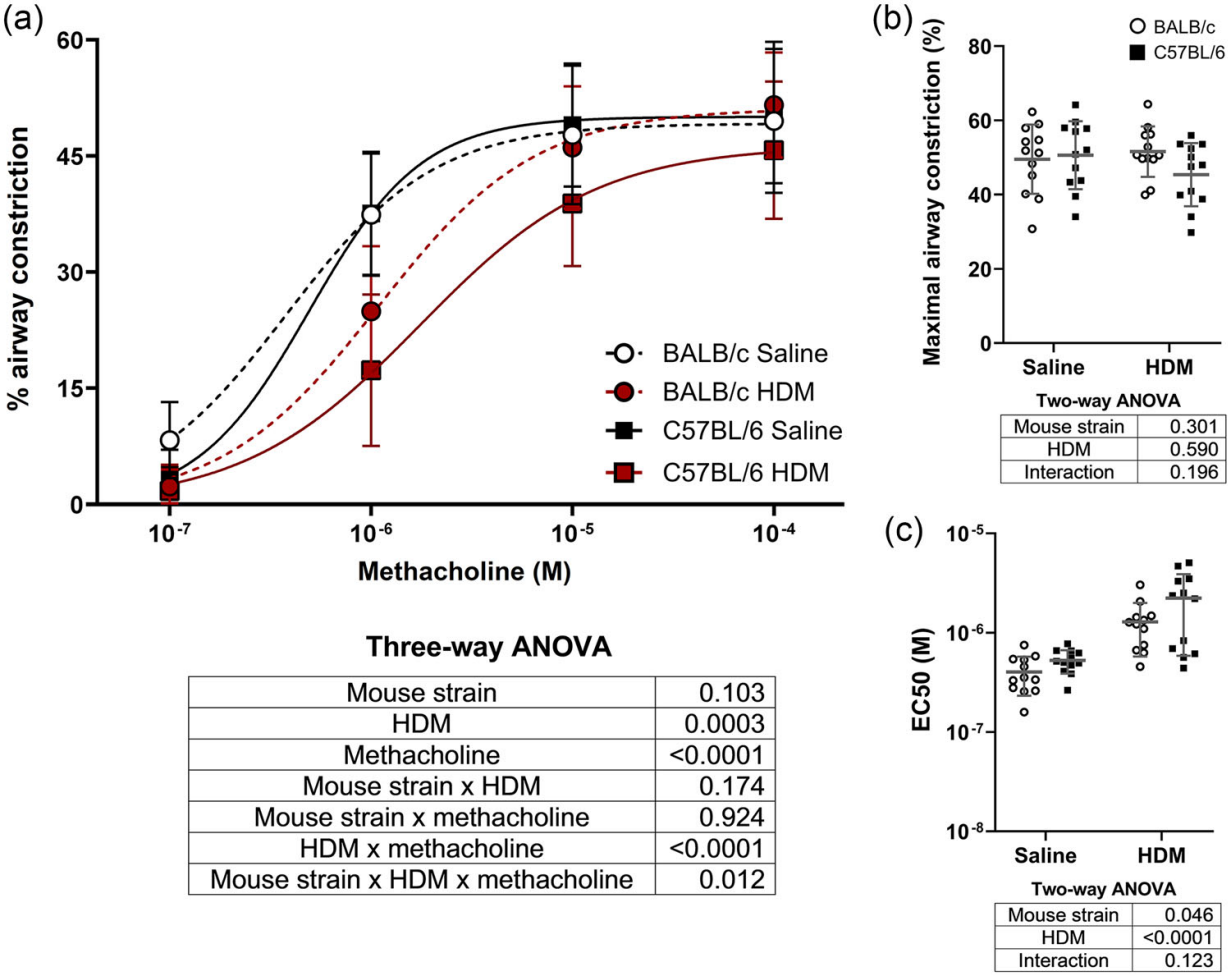
(a) Airway constriction in lung slices of BALB/c mice (circles) and C57BL/6 mice (squares) that were exposed to saline (black) or HDM (red) in response to increasing concentrations of methacholine. Each point on the curve represents the mean ± SD per group. The results of the three‐way ANOVA are provided below the graph. (b, c) The maximal airway constriction (b), representing the constriction in response to the highest concentration of methacholine tested (10^−4^ M), and the EC_50_ (c), representing the concentration of methacholine causing 50% of the maximal response, are shown as individual results, together with the mean ± SD in each group. The results of two‐way ANOVAs are also shown underneath each graph. *n* = 12 mice per group. Abbreviation: HDM, house dust mite.

Airway constriction in response to methacholine in lung slices is depicted in Figure 4. Twenty‐five to 62 airways were analysed per mouse (average of 44.8 ± 7.1). The HDM significantly affected airway constriction (Figure 4a). There was also a significant interaction between HDM and the concentration response to methacholine, suggesting that constriction was decreased by HDM at certain methacholine concentrations. In addition, there was a significant three‐way interaction between HDM, the mouse strain and the concentration response to methacholine, suggesting that the decreasing effect of HDM on the methacholine response at certain concentrations was greater in C57BL/6 than in BALB/c mice. Accordingly, the maximal constriction in response to methacholine was affected by neither HDM nor the mouse strain (Figure 4b), but the EC_50_ was increased by HDM and was higher in C57BL/6 than in BALB/c mice (Figure 4c).

**FIGURE 5:**
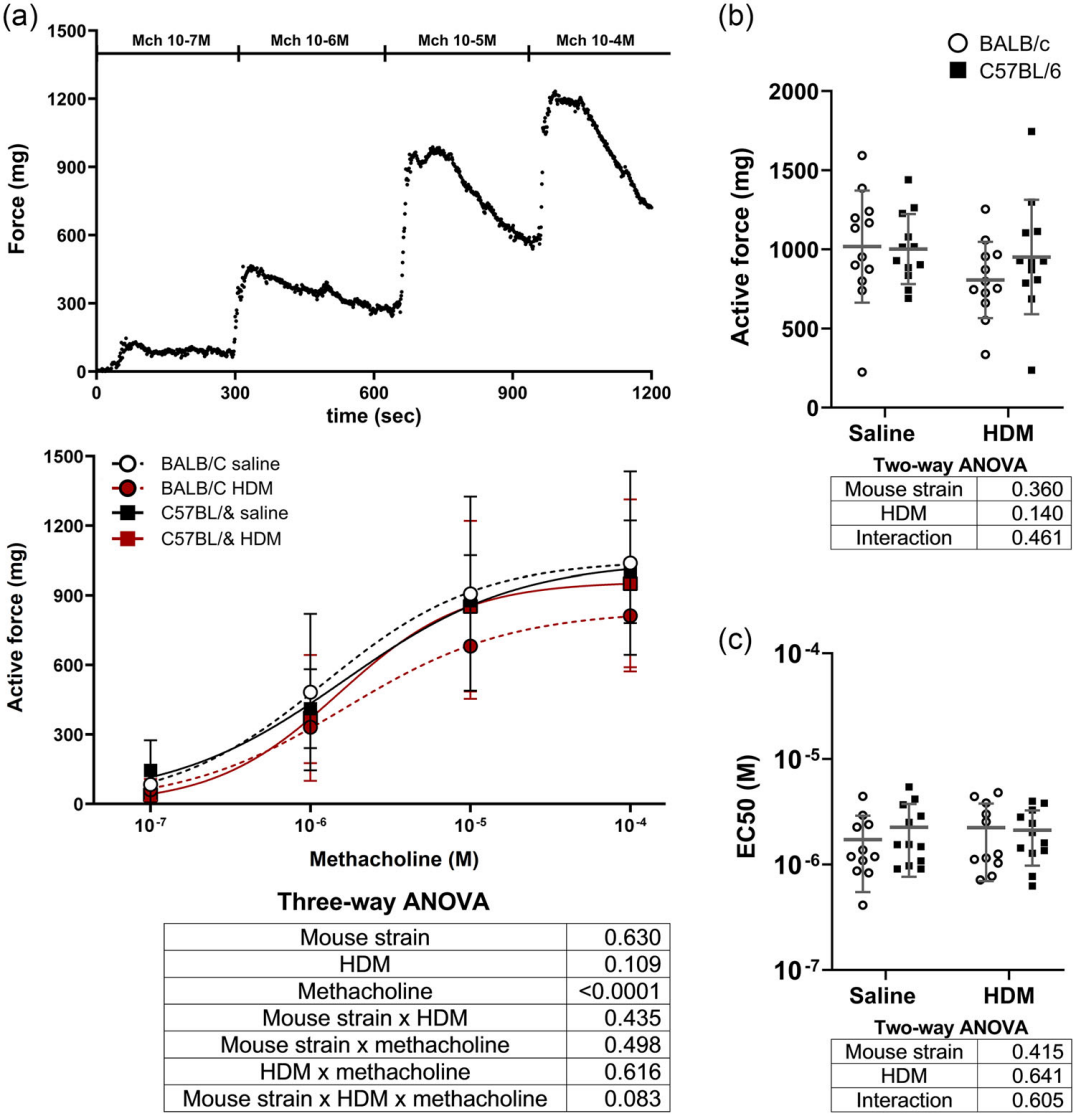
(a) The isometric force generated by excised tracheas of BALB/c mice (circles) and C57BL/6 mice (squares) that were exposed to either saline (black) and HDM (red) in response to increasing concentrations of methacholine. A representative trace (top), the mean ± SD in each group (middle) and the results of the three‐way ANOVA (bottom) are shown. (b, c) The maximal active force (b) and the EC_50_ (c), representing the concentration of methacholine causing 50% of the maximal response, are also shown as individual results, together with the mean ± SD in each group. The results of two‐way ANOVAs are also shown underneath each graph. *n* = 12 mice per group. Abbreviation: HDM, house dust mite.

**FIGURE 6:**
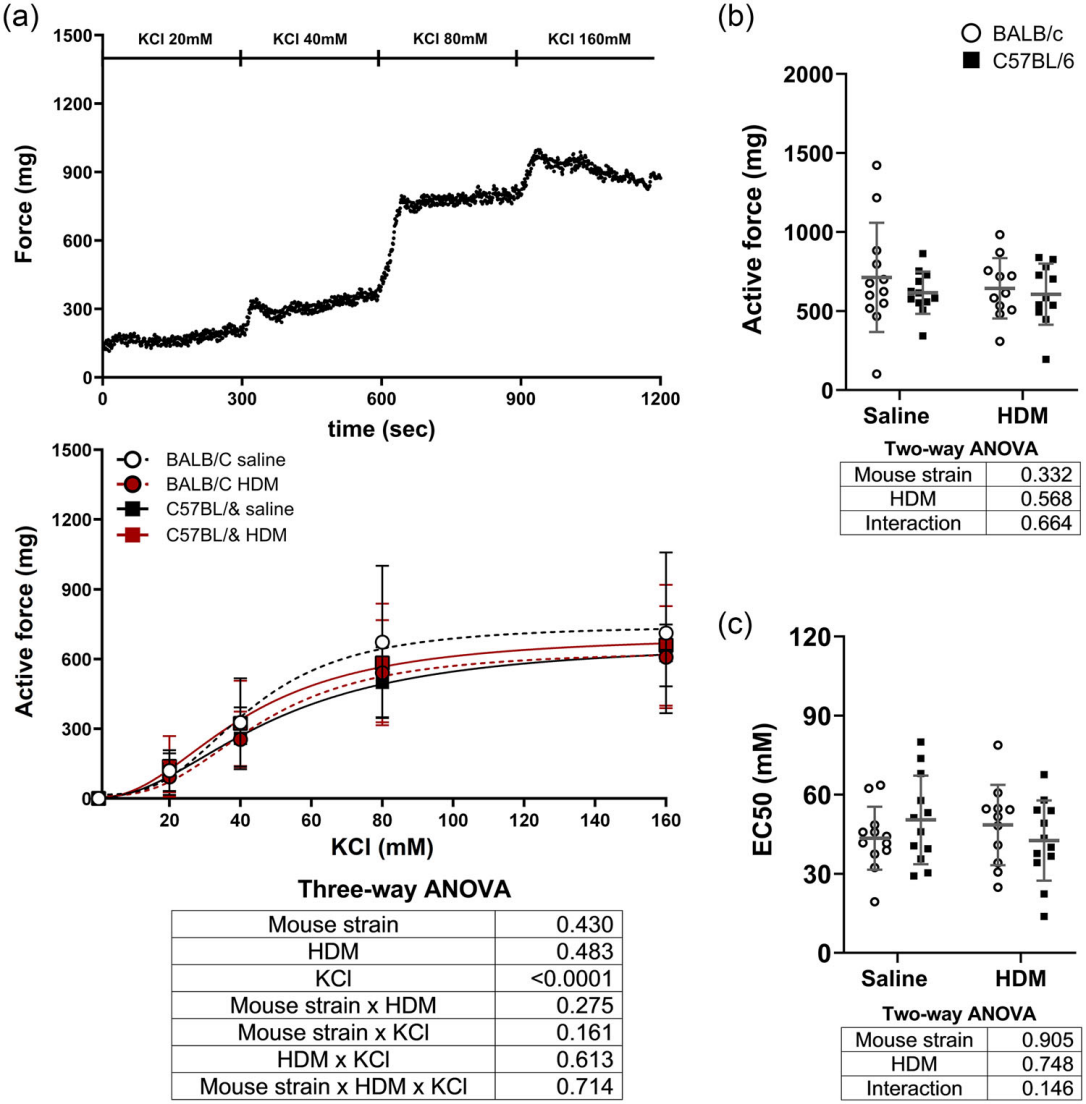
(a) The isometric force generated by excised tracheas of BALB/c mice (circles) and C57BL/6 mice (squares) that were exposed to either saline (black) and HDM (red) in response to increasing concentrations of KCl. A representative trace (top), the mean ± SD in each group (middle) and the results of the three‐way ANOVA (bottom) are shown. (b, c) The maximal active force (b) and the EC_50_ (c), representing the concentration of KCl causing 50% of the maximal response, are also shown as individual results, together with the mean ± SD in each group. The results of two‐way ANOVAs are also shown underneath each graph. *n* = 12 mice per group. Abbreviation: HDM, house dust mite.

The isometric force generated by excised tracheas in response to increasing concentrations of methacholine is depicted in Figure 5. The concentration–response curves were affected by neither HDM nor the mouse strain (Figure 5a). The maximal active force (Figure 5b) and the methacholine potency (EC_50_) (Figure 5b) were also not affected by HDM or the mouse strain. Similar results were obtained with KCl (Figure 6).

## 4 DISCUSSION

This study confirmed that BALB/c mice are more responsive to nebulized methacholine than C57BL/6 mice (Berndt et al., 2011; Duguet et al., 2000; Held & Uhlig, 2000; Leme et al., 2010; Levitt & Mitzner, 1989). This interstrain difference was especially striking in a model of experimental asthma induced by repeated exposures to HDM, which is also in line with results of previous studies (Adler et al., 2004; Boucher et al., 2021; De Vooght et al., 2010; Evans et al., 2015; Ewart et al., 2000; Gueders et al., 2009; Hirota, Ask et al., 2009; Kelada et al., 2011; Kenyon et al., 2003; Koya et al., 2006; Li et al., 2017; Sahu et al., 2010; Shinagawa & Kojima, 2003; Takeda et al., 2001; Van Hove et al., 2009; Zhang et al., 1997; Zhu & Gilmour, 2009). The novelty in the present study is that the in vivo hyperresponsiveness acquired in this specific, widely used (Carroll et al., 2023) model of asthma was not associated with a corresponding increase in ex vivo airway constriction. This dissociation between the in vivo and the ex vivo response to methacholine was shown in two mouse strains.

### 4.1 Interstrain difference in responsiveness

As already mentioned, it is well established that BALB/c mice are more responsive to nebulized methacholine than C57BL/6 mice (Berndt et al., 2011; Duguet et al., 2000; Held & Uhlig, 2000; Leme et al., 2010; Levitt & Mitzner, 1989). The in vivo results in the present study are thus consistent with these previous findings.

Comparisons of in vitro airway responsiveness between these two mouse strains were also undertaken previously. However, the results were inconsistent. Some studies with excised tracheas (Boucher et al., 2021) and lungs (Landgraf & Jancar, 2008) suggested that airway responsiveness in C57BL/6 mice is greater than that of BALB/c mice. Another study demonstrated no interstrain differences in isometric contraction of excised tracheas in response to three different spasmogens (carbachol, bradykinin and serotonin; Safholm et al., 2011). In contrast, some studies with lung slices suggested that airway constriction in BALB/c mice is not only greater (Duguet et al., 2000; Zeng et al., 2023) but also faster (Duguet et al., 2000) than in C57BL/6 mice. The reason why these results were not reproduced in the present study is not entirely clear. It is worth mentioning that the initial calibre of airways was greater in lung slices derived from BALB/c mice compared with the ones from C57BL/6 mice (data not shown). Given that constriction in the present study was expressed as a percentage of the initial calibre, it is likely that the absolute amount of airway constriction in BALB/c mice was greater than in C57BL/6 mice, which would then be consistent with the study by Duguet et al. (2000).

### 4.2 Responsiveness in experimental asthma

Interestingly, some investigators could induce ex vivo hyperresponsiveness repeatedly in a mouse model of allergic inflammation (Chiba et al., 2020, 2005). They used the main bronchi of male BALB/c mice sensitized and challenged with ovalbumin. Given that many early studies conducted with both mice without experimental asthma (Weinmann et al., 1990) and non‐asthmatic humans (Armour, Black et al., 1984; Armour, Lazar et al. 1984; Cerrina et al., 1986; de Jongste et al., 1988; Roberts et al., 1987; Taylor et al., 1985; Thomson, 1987) have demonstrated that the level of in vitro airway responsiveness rarely matches the degree of in vivo responsiveness, these results are surprising. They suggest that the choice of the mouse model of asthma (HDM vs. ovalbumin) or tissue preparation (trachea vs. bronchi) might influence the presence or absence of ex vivo hyperresponsiveness (Chiba et al., 2004).

In the present study, the hyperresponsiveness to nebulized methacholine was not associated with a corresponding increase in ex vivo airway constriction. This was true for both strains of mice. In fact, experimental asthma was associated with a decreased sensitivity to methacholine (i.e., an increased EC_50_) in airways of lung slices from both mouse strains. This is consistent with our recent study (Boucher et al., 2024). The origin of this reduced sensitivity is uncertain. It is potentially attributable to a real decrease in the sensitivity of the ASM. Another possibility is that the lung tissue (and/or the airway wall) might be stiffer in HDM‐ versus saline‐exposed mice (Gill et al., 2023; Rojas‐Ruiz et al., 2024). More force (and a higher concentration of methacholine) would thus be required to reach any level of constriction, explaining the decreased sensitivity. Another possibility might be related to a technical artefact. Given that there is already more fluid in the lungs of mice with experimental asthma (confirmed herein by heavier lungs; Figure 1B), pushing the same amount of agarose into their lungs might yield greater filling (agarose plus the exudative, inflammatory and mucosal fluids). This might then stiffen the lung tissue surrounding airways in lung slices and, consequently, decrease sensitivity by increasing the load, impeding ASM shortening. In other mouse models of asthma, ex vivo airway constriction in lung slices was decreased (Donovan et al., 2013), not affected (Bourke et al., 2019; Kim et al., 2015) or increased (Liu et al., 2017). More studies will be needed, perhaps combined with traction force microscopy to track the changes in stress and strain within and around the airway wall during constriction.

Complementary ex vivo studies were conducted with excised tracheas. Given that the trachea contracted in isometric conditions in these assays, its responsiveness cannot be influenced by lung tissue stiffness (unlike airway constriction in lung slices). These experiments were especially important to tease out whether a change in ASM force or a change in lung tissue mechanical properties is at the origin of the decreased methacholine sensitivity seen in airways from HDM‐exposed mice. The results showed no signs of excessive isometric force from tracheas isolated from HDM‐exposed mice in both mouse strains. This was shown in response to the classical spasmogen methacholine, which signals via its cognate muscarinic 3 receptor (M3R). It was also shown in response to KCl, a spasmogen acting independently from G protein‐coupled receptor signalling. Inasmuch as the contraction of the ASM from the trachea is representative of ASM contraction in smaller airways (Gunst & Stropp, 1988), this suggests that an increased lung tissue stiffness (rather than a defect in ASM force) is likely to account for the decreased methacholine sensitivity of airway constriction seen in lung slices of HDM‐exposed mice. Another hint supporting this conjecture is the significant difference in the sensitivity of airway constriction between mouse strains. Indeed, airways from C57BL/6 mice were less sensitive to the constricting effect of methacholine than the ones from BALB/c mice (Figure 4C; *P* = 0.046), and it was recently shown that the former have stiffer lung tissue than the latter (Rojas‐Ruiz et al., 2023). One downside of the experiments conducted with isolated tracheas in the present study is that the level of inflammation was not quantified specifically in the tracheal tissue, and the presence versus the absence of inflammation in these types of preparations was previously associated with ex vivo hyperresponsiveness, both in humans (Ijpma et al., 2020) and in a different mouse model of asthma (Chiba et al., 2004).

The most likely cause of in vivo hyperresponsiveness in the mouse model of asthma presented herein is small airway closure. In another acute mouse model of asthma induced by ovalbumin sensitization and challenge, computational modelling has clearly demonstrated that an increased propensity for small airway closure, attributable to a thicker airway wall (caused by oedema, for example) and the accumulation of inflammatory fluid within the airway lumen, is sufficient to explain hyperresponsiveness to nebulized methacholine (Wagers et al., 2004). In other words, a normal level of methacholine‐induced ASM contraction in inflamed lungs with an increased propensity for airway closure was sufficient to provoke exaggerated changes in lung mechanics (Wagers et al., 2004). Although ASM contraction was not measured directly in that study, it was subsequently shown that airway closure caused by nebulized methacholine was manifestly greater in this mouse model of asthma (Lundblad et al., 2007). Based on these findings, we surmise that in vivo hyperresponsiveness in the mouse model presented in this study is mainly attributable to excessive airway closure.

The dissociation between the effect of experimental asthma on the in vivo and ex vivo response to methacholine in the present study is reminiscent to findings in humans with and without asthma. Despite methacholine hyperresponsiveness in vivo being a pathognomonic feature of human asthma, there is a dearth of evidence, as in mice, to support its association with an enhanced ex vivo contractility of the ASM (Chin et al., 2012; Ijpma et al., 2015, 2020; Noble et al., 2013; Whicker et al., 1988; reviewed by Wright et al., 2013). There are a few reports showing significant differences in some ASM contractile properties of asthmatics compared with non‐asthmatics, such as a decreased sensitivity (Ijpma et al., 2015, 2020; Whicker et al., 1988), an increased stress‐generating capacity (Ijpma et al., 2020), increased airway narrowing (Noble et al., 2013) and an improved ability to tolerate the decline in contractility caused by oscillating strains simulating breathing manoeuvres (Chin et al., 2012). However, the reported alterations are inconsistent between studies. Notably, most of these studies were testing several contractile properties with small sample sizes, which are the perfect ingredients for type 1 errors, especially while considering the inherent variability observed with these types of preparations. In our opinion, and consistent with the present mouse study, the bulk of studies published so far with human tissues suggest that excessive ASM contraction is unlikely to be an important or common contributor to in vivo hyperresponsiveness. As in mice (Lundblad et al., 2007; Wagers et al., 2004) and as reported in several studies (Chapman et al., 2008; Dame Carroll et al., 2015; Farrow et al., 2012), a main cause of in vivo hyperresponsiveness in human asthma is airway closure.

One possibility in human asthma is that contraction might be excessive owing to ASM enlargement. This was the conclusion drawn by Noble et al. (2013) in a study with human airways. More precisely, they have shown that the stress‐generating capacity of asthmatic ASMs are not different from ASMs derived from non‐asthmatics, but that the increased amount of ASM still leads to more airway narrowing (Noble et al., 2013). This is a very important finding that needs to be investigated further, because a larger amount of ASM is a common feature of asthmatic airways (Elliot et al., 2015). Given that our acute mouse model of asthma does not exhibit ASM enlargement, this might be an additional reason why there was no excessive airway constriction in the present study. Yet, these models of acute allergic lung inflammation induced by HDM are widely used to study experimental asthma (Carroll et al., 2023). We think it is important to be aware that these models do not exhibit excessive airway constriction (at least not ex vivo).

In addition, the results of the present study do not preclude the existence of in vivo factors that might render the ASM hypercontractile when it operates in an inflamed, ‘asthmatic’ milieu. Owing to the phenomenal contractile plasticity of the ASM (Auger et al., 2016; Black et al., 2012; Bossé et al., 2008; Gunst et al., 1995; Halayko et al., 2008; Hirota, Nguyen et al., 2009), it is possible that the tissue reverts to a normal contractile state when isolated and studied ex vivo (away from all in vivo asthmatic factors that might be enhancing its contractility). Safholm et al. (2011), for example, have shown that incubating tracheal rings from BALB/c mice with some inflammatory mediators increases the contractile response to certain spasmogens, which was not the case for tracheal rings from C57BL/6 mice. They suggested that the greater susceptibility of BALB/c mice to the development of in vivo hyperresponsiveness in the context of experimental asthma might stem, at least in part, from this acquired hypercontractility (Safholm et al., 2011). Notably, nearly a hundred different molecular mediators have been shown to alter contractility in other preparations of ASM (Gazzola et al., 2020).

The dissociation between the in vivo and the ex vivo response to methacholine can also rely on an interplay between the ASM and other lung tissues. For example, *Der p 1*, the immunodominant allergen from *D. pteronyssinus* species found in HDM, was shown to reduce the lung expression of several G protein‐coupled receptors involved in ASM contraction, including the muscarinic 2 receptor (M2R) (Kelada et al., 2011). Although airway constriction is mediated mainly by the M3R (Fisher et al., 2004; Roffel et al., 1990; Struckmann et al., 2003), M2R is required for optimal contraction of murine airways (Alkawadri et al., 2022; Struckmann et al., 2003). Counterintuitively, M2R‐deficient mice are hyperresponsive to intravenous administration of methacholine (Fisher et al., 2004). This has been attributed to the prejunctional role of the M2R in limiting acetylcholine release from the nerves (Fryer & Jacoby, 1998). In fact, a dysfunctional M2R was repeatedly shown to contribute to in vivo hyperresponsiveness in animal models of asthma induced by a variety of offending triggers, such as antigens, viruses, ozone and others (Fryer & Jacoby, 1998; Rynko et al., 2014). An unfettered release of acetylcholine is likely to increase ASM tone and, consequently, ASM force (Bossé et al., 2009), and this effect might then be greater than the postjunctional role of the M2R in potentiating the contraction of ASM (Alkawadri et al., 2022; Struckmann et al., 2003). Therefore, the decreased expression of M2R alone caused by HDM (Kelada et al., 2011) might be one putative molecular mechanism whereby mice with experimental asthma in our study are hyperresponsive in vivo, whereas their airway constriction in lung slices is attenuated at low methacholine concentrations. However, unless the downregulation of M2R (Kelada et al., 2011) or the potentiation of ASM contraction by the M2R (Alkawadri et al., 2022; Struckmann et al., 2003) is specific to peripheral airways (and not the trachea), this would not explain the lack of differences in methacholine sensitivity between excised tracheas of HDM‐ and saline‐exposed mice.

## 5 CONCLUSION

It is concluded that, at least in the specific mouse model used in the present study, the hyperresponsiveness to nebulized methacholine caused by experimental asthma is not matched by a corresponding increase in ex vivo airway constriction. In our opinion, the most likely explanation is that a normal level of ASM shortening is sufficient to trigger in vivo hyperresponsiveness when it operates in combination with other lung alterations seen in mice with experimental asthma (Bossé, 2021; Bossé et al., 2010; Wagers et al., 2004).

## AUTHOR CONTRIBUTIONS

Andrés Rojas‐Ruiz, Magali Boucher, Cyndi Henry, Percival Graham, Jorge Soliz and Ynuk Bossé contributed to the development of the experimental design. Andrés Rojas‐Ruiz, Magali Boucher, Cyndi Henry, Rosalie Packwood and Louis Gélinas performed laboratory experiments. Andrés Rojas‐Ruiz, Magali Boucher and Ynuk Bossé analysed the data. Ynuk Bossé wrote the first version of the manuscript. All authors edited the manuscript, read and approved the final version of the manuscript and agree to be accountable for all aspects of the work in ensuring that questions related to the accuracy or integrity of any part of the work are appropriately investigated and resolved. All persons designated as authors qualify for authorship, and all those who qualify for authorship are listed.

## CONFLICT OF INTEREST

P.G. is employed by SCIREQ Inc., a commercial entity with interests in topics related to the content of the present work. Y.B. holds an operating grant in partnership with SCIREQ Inc. A.R.R., M.B., C.H., L.G., R.P. and J.S. have no conflict of interest.

## Data Availability

The datasets used and analysed during the present study are available from the corresponding author on reasonable request.
